# Why Intergenerational Connections Matter in Higher Education

**DOI:** 10.1093/geroni/igaa057.1772

**Published:** 2020-12-16

**Authors:** Joann Montepare, Skye Leedahl

**Affiliations:** 1 Lasell University, Newton, Massachusetts, United States; 2 University of Rhode Island, Kingston, Rhode Island, United States

## Abstract

The Academy for Gerontology in Higher Education (AGHE) has a long and rich history of fostering the commitment of higher education to the field of aging through education, research, and public service. To this end, AGHE members have made innovative and effective use of intergenerational strategies. This presentation will highlight these efforts gleaned from AGHE’s official journal Gerontology and Geriatrics Education and Intergenerational Learning, Research, and Community Engagement interest group (ILRCE), with special attention to intergenerational classroom exchange, service-learning and community activities, and socially-focused undertakings. Contributions to the evaluation of intergenerational programs will also be noted. The presentation will conclude with a discussion of how this work is now helping to shape the Age-Friendly University (AFU) initiative which calls for institutions of higher education to promote intergenerational learning to facilitate the reciprocal sharing of expertise between learners of all ages.

